# Characterization and Identification of Variations in Types of Primary Care Visits Before and During the COVID-19 Pandemic in Catalonia: Big Data Analysis Study

**DOI:** 10.2196/29622

**Published:** 2021-09-14

**Authors:** Francesc Lopez Segui, Guillem Hernandez Guillamet, Héctor Pifarré Arolas, Francesc X Marin-Gomez, Anna Ruiz Comellas, Anna Maria Ramirez Morros, Cristina Adroher Mas, Josep Vidal-Alaball

**Affiliations:** 1 Centre de Recerca en Economia i Salut Pompeu Fabra University Barcelona Spain; 2 Gerència Territorial de la Catalunya Central Institut Català de la Salut Sant Fruitós de Bages Spain; 3 Health Promotion in Rural Areas Research Group Gerència Territorial de la Catalunya Central, Institut Català de la Salut Sant Fruitós de Bages Spain; 4 Unitat de Suport a la Recerca de la Catalunya Central Fundació Institut Universitari per a la recerca a l'Atenció Primària de Salut Jordi Gol i Gurina Sant Fruitós de Bages Spain; 5 Faculty of Medicine University of Vic Central University of Catalonia Vic Spain; 6 Centre d’Atenció Primària Sant Joan de Vilatorrada Gerència Territorial de la Catalunya Central Institut Català de la Salut Sant Fruitós de Bages Spain; 7 Sant Joan de Déu Hospital Barcelona Spain

**Keywords:** COVID-19, primary care, diagnose variations, big data, ICD10, health system, big data, primary care, healthcare system

## Abstract

**Background:**

The COVID-19 pandemic has turned the care model of health systems around the world upside down, causing the abrupt cancellation of face-to-face visits and redirection of the model toward telemedicine. Digital transformation boosts information systems—the more robust they are, the easier it is to monitor the health care system in a highly complex state and allow for more agile and reliable analysis.

**Objective:**

The purpose of this study was to analyze diagnoses from primary care visits and distinguish between those that had higher and lower variations, relative to the 2019 and 2020 periods (roughly before and during COVID-19), to identify clinical profiles that may have been most impaired from the least-used diagnostic codes for visits during the pandemic.

**Methods:**

We used a database from the Primary Care Services Information Technologies Information System of Catalonia. We analyzed the register of visits (n=2,824,185) and their *International Classification of Diseases* (*ICD-10*) diagnostic codes (n=3,921,974; mean 1.38 per visit), as approximations of the reasons for consultations, at 3 different grouping levels. The data were represented by a term frequency matrix and analyzed recursively in different partitions aggregated according to date.

**Results:**

The increase in non–face-to-face visits (+267%) did not counterbalance the decrease in face-to-face visits (–47%), with an overall reduction in the total number of visits of 1.36%, despite the notable increase in nursing visits (10.54%). The largest increases in 2020 were visits with diagnoses related to COVID-19 (*ICD-10* codes Z20-Z29: 2.540%), along with codes related to economic and housing problems (*ICD-10* codes Z55-Z65: 44.40%). Visits with most of the other diagnostic codes decreased in 2020 relative to those in 2019. The largest reductions were chronic pathologies such as arterial hypertension (*ICD-10* codes I10-I16: –32.73%) or diabetes (*ICD-10* codes E08-E13: –21.13%), but also obesity (E65-E68: –48.58%) and bodily injuries (*ICD-10* code T14: –33.70%). Visits with mental health–related diagnostic codes decreased, but the decrease was less than the average decrease. There was a decrease in consultations—for children, adolescents, and adults—for respiratory infections (*ICD-10* codes J00-J06: –40.96%). The results show large year-on-year variations (in absolute terms, an average of 12%), which is representative of the strong shock to the health system.

**Conclusions:**

The disruption in the primary care model in Catalonia has led to an explosive increase in the number of non–face-to-face visits. There has been a reduction in the number of visits for diagnoses related to chronic pathologies, respiratory infections, obesity, and bodily injuries. Instead, visits for diagnoses related to socioeconomic and housing problems have increased, which emphasizes the importance of social determinants of health in the context of this pandemic. Big data analytics with routine care data yield findings that are consistent with those derived from intuition in everyday clinical practice and can help inform decision making by health planners in order to use the next few years to focus on the least-treated diseases during the COVID-19 pandemic.

## Introduction

**Figure 1:**
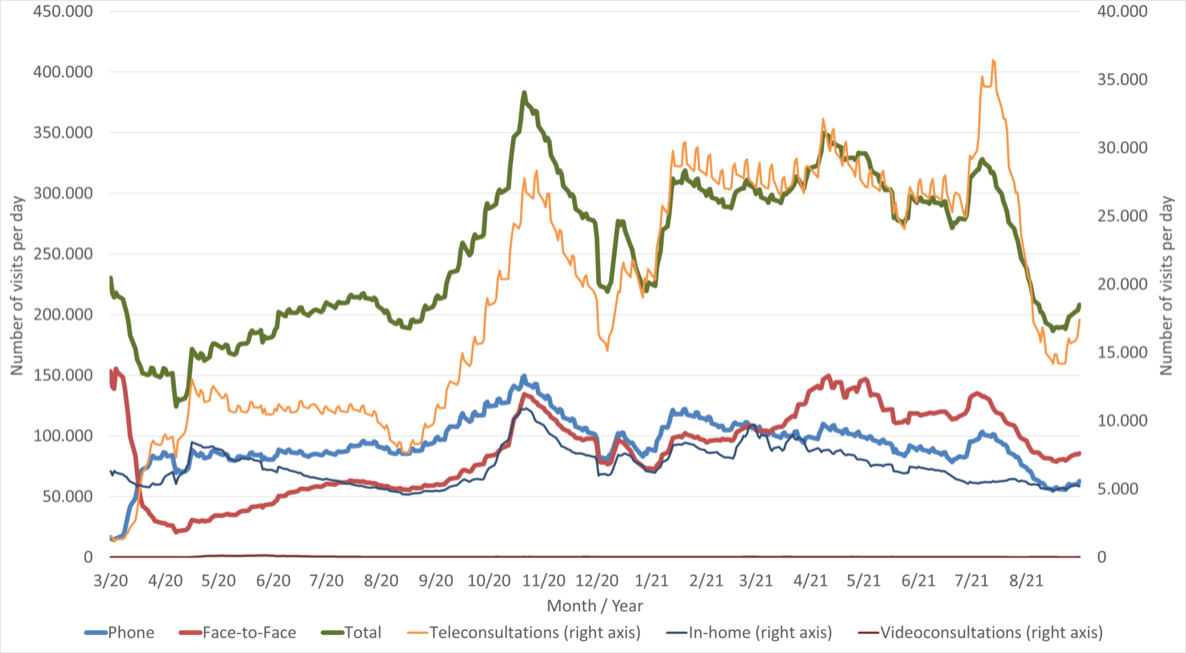
A 7-day rolling average (with weekends and holidays not included) of primary care daily visits in Catalonia, by type, from March 2020 to August 2021 (unpublished data, Catalan Ministry of Health).

The COVID-19 pandemic has turned the care model of health systems around the world upside down, with the abrupt cancellation of face-to-face visits, redirection of the model toward non–face-to-face care, and then the gradual recovery of services, first for priority population sectors and later for the general population [1-4]. Telemedicine in primary care in Catalonia includes provider–patient or provider–provider communications and can take place synchronously (telephone or video calls) or asynchronously (teleconsultations). By the end of February 2021, all face-to-face activity had not yet recovered, although the total number of visits was approximately 15% higher than that at the start of the pandemic (Figure 1), in part because of the increasing use of telemedicine (mostly in the form of telephone consultations).

Because of the disruptions, health systems have begun to identify population profiles that may be most adversely affected by changes and underdiagnosed diseases during the pandemic [5-8]. Some patients have had to wait longer than before to receive health care. It has been observed, for example, that cardiac arrests outside hospital have increased, with patients having worse prognoses [9], or that patients with heart failure have had fewer admissions, but when admitted, heart failure has been more severe and admission to the intensive care unit is required more often [10]. It has also been noted that access to telemedicine visits has not been the same for the whole population and that, when this type of care becomes predominant, inequalities in access to care may arise [11,12].

According to a recent study [13], COVID-19 has resulted in loss of years of life over 7 times greater than that in a typical influenza season in Spain. The health emergency has precipitated policy maker decision-making that has often been without any existing evidence and with long delays [14], but it has also highlighted the value of information systems and, more generally, the role of health policy evaluation agencies [15]. Robust information systems make it easier to assess and monitor the health system in a highly complex state and allow for agile and reliable analysis. In this context, we explored the potential for the use of a massive amount of primary care data from the Catalan public health system to inform quick, granular decision making regarding which areas have been most neglected as a result of the change in the care model. Specifically, we aimed to identify which diagnoses in primary care have increased and decreased from 2019 to 2020 (approximately before and during the COVID-19 pandemic), in order to determine which areas need to be strengthened in view of the gradual recovery of health system activity.

## Methods

### Ethics

The study was conducted according to the guidelines of the Declaration of Helsinki and in accordance with European General Data Protection Regulations and the Spanish Organic Law on Data Protection and Guarantee of Digital Rights. No ethical approval was required; analyses were conducted only on aggregated data.

### Data

The Catalan health system provides universal coverage to 7.6 million inhabitants. Administratively, it is composed of a single public payer and several publicly or privately owned service providers, in an integrated system and with an important role of community and primary health care and the use of information technology. We used a database from the Primary Care Services Information Technologies System of the Health Region of Central Catalonia (Catalonia, Spain) belonging to the Catalan Institute of Health. In this region, the Health Information System is used by the majority (32/37, 86.48%) of Primary Care centers, which facilitated analysis. The variable of interest was primary care visits according to the *International Statistical Classification of Diseases tenth revision* (*ICD-10*) [16] diagnostic codes. These codes were used as a proxy for the reason for the visit.

Data from primary care visits in 2020 (the period covering most of the COVID-19 pandemic) and 2019 (the period used as a comparator)—both face-to-face (at the primary care center or at home) and non–face-to-face (telemedicine tools including telephone and teleconsultations)—were analyzed. One visit could result in several diagnostic codes and not all visits were associated with at least one, as doing so is not mandatory in the Catalan primary care electronic health record. Consequently, the data set was reduced to visits in which at least one diagnosis had been identified.

### Statistical Analysis

Diagnostic codes attributed to visits were analyzed at 3 levels of aggregation: aggregation level 1 (21 codes, with format “Neoplasms, C00-D49”), which corresponds to the concept of *chapters* according to the reference *ICD-10* dictionary [16]; aggregation level 2, which was a second, more specific grouping (284 different codes, formatted “Malignant neoplasms of digestive organs, C15-C26”), which corresponded to the concept of *blocks* in the above-mentioned dictionary; and aggregation level 3, which was a third grouping with even more specificity (1532 different codes, with the format “Malignant neoplasm of anus and anal canal, C21”).

The variety of diagnostic codes used in clinical practice suggested that there would be a need for the use of big data analysis techniques. Visit data were recursively analyzed in 4 different partitions, and the diagnoses were aggregated according to date. *ICD-10* groupings were represented with a term frequency matrix [17,18], where matrix columns indicated the absolute number of each diagnostic code.

The variation index was used to represent the relative weight of the variation of a diagnostic code over the absolute total interannual variation of all codes. Thus, in addition to the frequency of use of each diagnostic code, this index takes into account the importance of each on a linear scale to allow visualization of the diagnoses on 2 scales (code frequency and variation index). Data were analyzed using R (version 3.4.3; R Foundation for Statistical Computing).

## Results

**Figure 2:**
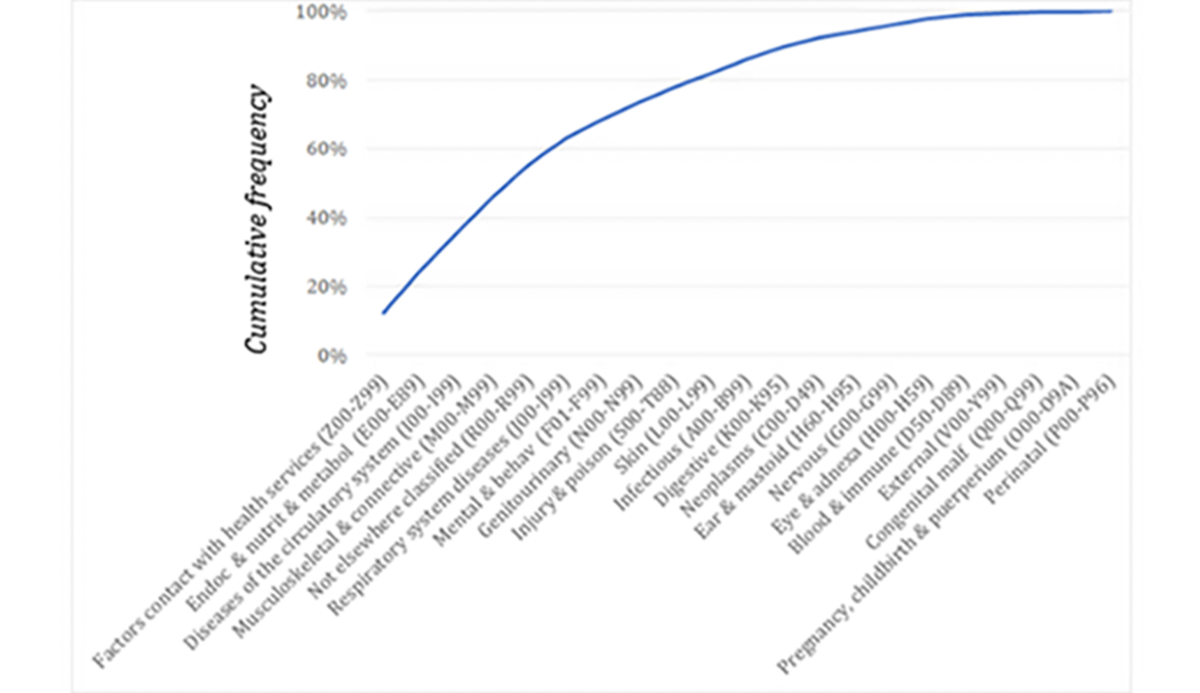
Aggregation level 1 cumulative frequency of the 21 most used *International Statistical Classification of Diseases, tenth revision*, diagnostic codes (2019 and 2020). behav: behavioral, endoc: endocrine, malf: malformation, metabol: metabolic, nutrit: nutritional.

**Figure 3:**
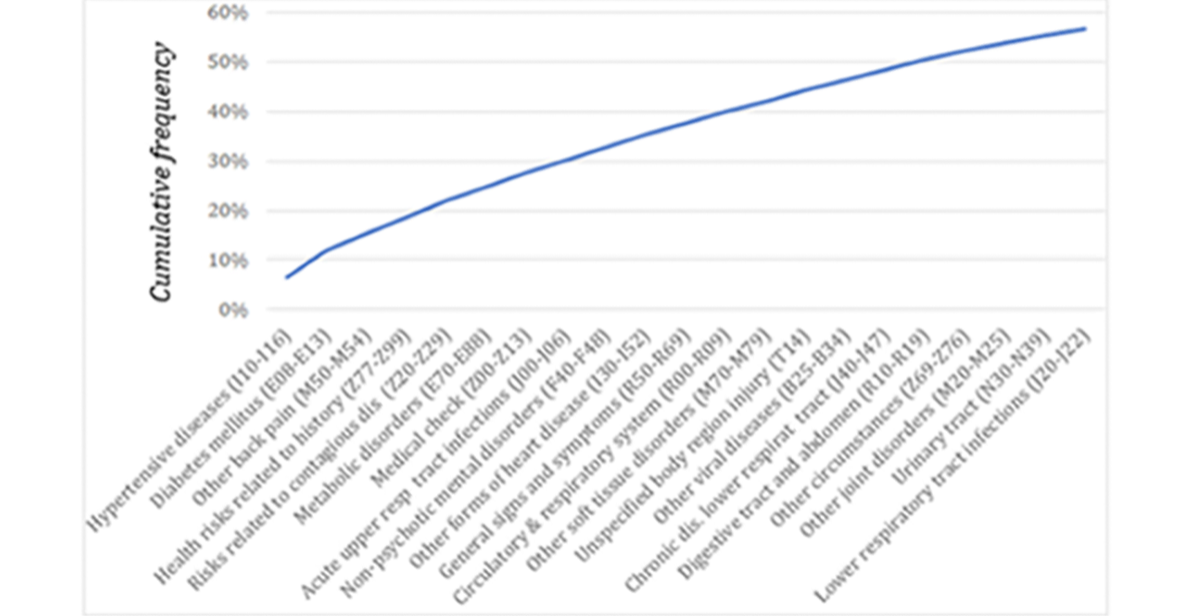
Aggregation level 2 cumulative frequency of the 21 most used *International Statistical Classification of Diseases, tenth revision*, diagnostic codes (2019 and 2020). dis: disease, resp/respirat: respiratory.

There were 3,555,799 primary care visits for 376,486 citizens (a total of 404,245 citizens are in the reference population of this health region) in 2019 and 2020, and at least one diagnosis was identified in 79.43% of visits (2824371/3,555,799). The degree of coding is slightly higher in the face-to-face visits compared to the non-face-to-face visits Regarding the effects of the pandemic on missing data, during 2019, 18.46% (262,460/1,421,779) of visits had no associated diagnoses, while in 2020, missing instances reached 22.50% (315,541/1,402,406), representing a limited impact on reported outcomes. The final database was composed of 2,824,185 visits for 358,419 different citizens, with 3,921,974 diagnostic visit codes (1.38 codes per visit on average; 3.5% of visits had more than 3 associated codes; Figure 2 and Figure 3).

The total number of visits in 2020 decreased slightly (–1.36%) compared to those in 2019; if COVID-19–related visits are excluded, this figure is even lower (–7.56%). The type of visit changed markedly (Table 1). In 2019, most visits (1,212,800/1,421,779, 85.30%) were face-to-face, while in 2020 more than half of visits (768,548/1,402,406, 54.80%) were telemedicine. The increase in this type of visit, therefore, has not been able to compensate for the decrease in face-to-face visits.

**Table 1:** Evolution of visits.

Characteristic			2019		2020		Difference, %
Total patients attended			287,936		287,965		0.01
**Total visits, n**			1,421,779		1,402,406		–1.36
	Face-to-face, n (%)	1,212,800 (85.30)		633,858 (45.20)		–47.74	
	Telemedicine, n (%)	208,979 (14.70)		768,548 (54.80)		267.76	
	General medicine, n (%)	859,153 (60.43)		822,433 (58.64)		–4.27	
	Nursing, n (%)	427,959 (30.10)		473,051 (33.73)		10.54	
	Pediatrics, n (%)	134,667 (9.47)		106,922 (7.62)		–20.60	
	Early childhood^a^, n (%)	95,098 (6.69)		76,460 (5.45)		–24.38	
	Childhood^b^, n (%)	57,436 (4.04)		52,101 (3.72)		–10.24	
	Adolescence^c^, n (%)	55,768 (3.92)		61,693 (4.40)		9.60	
	Youth^d^, n (%)	55,943 (3.93)		69,287 (4.94)		19.26	
	Adulthood^e^, n (%)	475,366 (33.43)		514,327 (36.67)		7.58	
	Older adult^f^, n (%)	682,168 (47.98)		628,538 (44.82)		–8.53	
Total diagnostic codes, n			2,079,680		1,912,342		–10.57
Diagnostic codes per visit, mean (SD)			1.46 (1.00)		1.36 (0.89)		–6.84
**Sex, n (%)**							
	Male	641,602 (45.13)		627,806( 44.77)		–2.15	
	Female	780,074 (54.87)		774,486 (55.23)		–0.72	
Age of visits, mean (SD)			53.54 (26.25)		52.89 (25.72)		–1.21

^a^Early childhood: 0 to 5 years old.

^b^Childhood: infancy to 11 years old.

^c^Adolescence: 12 to 18 years old.

^d^Youth: 14-26 years old.

^e^Adulthood: 27 to 59 years old.

^f^Older adult: over 60 years old.

In relation to the specialty, the number of visits with doctors decreased slightly (–4.27%), the number of visits with nurses increased (10.54%), and the number of pediatric visits decreased (–20.60%). Despite this decrease, the mean age of patients decreased by approximately 1 year (from 53.54 in 2019 to 52.89 years in 2020). There was a variation in visits according to age groups: there was a decrease in visits for patients in early childhood and infancy (from 0 to 5 years old), an increase in patients in adolescence (12 to 18 years old) to adulthood (27 to 59 years old), and a decrease in older adult patients. The proportion of visits made by women was 55.04% (1,554,560/2,824,185); the magnitude remained fairly unchanged between 2019 and 2020.

The cumulative frequency of the 21 most used diagnostic codes for the 2 first levels of grouping were analyzed (Figure 3). In the case of aggregation level 1, the 5 code groupings comprised more than half of the total number of codes (Factors influencing health status and contact with health services; Endocrine, nutritional, and metabolic diseases; Diseases of the circulatory system; Diseases of the musculoskeletal system and connective tissue; Signs, symptoms, and abnormal test results not classified elsewhere). For aggregation level 2, the 5 most-used codes comprised approximately one-quarter of the total (Hypertensive diseases; Diabetes mellitus; Other back disorders; Persons with potential health risks related to personal and family history and certain conditions influencing health status; Persons with potential health risks related to communicable diseases).

For aggregation level 1, large interannual variations were observed (Table 2), especially for diagnostic codes related to *factors influencing health status and contact with health services* (*ICD-10* codes Z00-Z99), codes that were initially used for diagnoses related to COVID-19, and the diagnosis of *certain infectious and parasitic diseases*, which nearly doubled in 2020. The rest of the codes decreased in 2020 compared to 2019; however, those related to mental health decreased less than the rest (*ICD-10* codes F01-F99: –3.39%). Diagnostic codes related to injuries were reduced by almost one-quarter (*ICD-10* codes S00-T88: –26.85%), possibly due to a drastic reduction in accidents resulting from people engaging in less activity during confinement. The variation index shows the weight of each of these variations on overall disruption (Table 3).

**Table 2:** Use of *International Statistical Classification of Diseases, tenth revision*, diagnostic codes (aggregation level 1).

Diagnostic code	Total, n (%)	2019, n (%)	2020, n (%)	Interannual variation, %	Variation index
Factors influencing health status and contact with health services (Z00-Z99)	483,368 (12.32)	186,604 (9.06)	296,764 (15.93)	59.03	0.22
Endocrine, nutritional, and metabolic diseases (E00-E89)	464,009 (11.83)	261,076 (12.68)	202,933 (10.90)	–22.27	0.11
Diseases of the circulatory system (I00-I99)	440,256 (11.23)	252,555 (12.26)	187,701 (10.08)	–25.68	0.13
Diseases of the musculoskeletal system and connective tissue (M00-M99)	405,813 (10.35)	224,901 (10.92)	180,912 (9.71)	–19.56	0.09
Signs, symptoms, and abnormal test results not elsewhere classified (R00-R99)	385,121 (9.82)	200,711 (9.75)	184,410 (9.90)	–8.12	0.03
Diseases of the respiratory system (J00-J99)	297,074 (7.57)	176,405 (8.57)	120,669 (6.48)	–31.60	0.11
Mental, behavioral, and neurodevelopmental disorders (F01-F99)	214,709 (5.47)	109,203 (5.30)	105,506 (5.66)	–3.39	0.01
Diseases of the genitourinary system (N00-N99)	182,642 (4.66)	96,692 (4.70)	85,950 (4.61)	–11.11	0.02
Injuries, poisonings, and other consequences of external causes (S00-T88)	182,045 (4.64)	105,135 (5.11)	76,910 (4.13)	–26.85	0.06
Diseases of the skin and subcutaneous tissue (L00-L99)	164,759 (4.20)	89,119 (4.33)	75,640 (4.06)	–15.12	0.03
Certain infectious and parasitic diseases (A00-B99)	151,999 (3.88)	52,220 (2.54)	99,779 (5.36)	91.07	0.09
Diseases of the digestive system (K00-K95)	143,793 (3.67)	78,968 (3.83)	64,825 (3.48)	–17.91	0.03
Neoplasms (C00-D49)	102,910 (2.62)	55,201 (2.68)	47,709 (2.56)	–13.57	0.01
Diseases of the ear and mastoid process (H60-H95)	81,495 (2.08)	47,699 (2.32)	33,796 (1.81)	–29.15	0.03
Diseases of the nervous system (G00-G99)	72,150 (1.84)	38,057 (1.85)	34,093 (1.83)	–10.42	0.01
Diseases of the eye and adnexa (H00-H59)	61,810 (1.58)	36,837 (1.79)	24,973 (1.34)	–32.21	0.02
Diseases of the blood and blood-forming organs and disorders affecting the immune mechanism (D50-D89)	45,551 (1.16)	25,305 (1.23)	20,246 (1.09)	–19.99	0.01
External causes of morbidity (V00-Y99)	23,659 (0.60)	12,481 (0.61)	11,178 (0.60)	–10.44	0.00
Congenital malformations. congenital deformities, and congenital chromosomal anomalies (Q00-Q99)	10,034 (0.26)	5493 (0.27)	4541 (0.24)	–17.33	0.00
Pregnancy. childbirth, and puerperium (O00-O9A)	7023 (0.18)	3788 (0.18)	3235 (0.17)	–14.60	0.00
Certain conditions originating in the perinatal period (P00-P96)	1754 (0.04)	991 (0.05)	763 (0.04)	–23.01	0.00

**Table 3:** Top 10 *International Statistical Classification of Diseases, tenth revision*, diagnostic codes according to the variation index.

Diagnostic code		Variation index
**Aggregation level 2**		
	Persons with potential health risks related to communicable diseases (Z20-Z29)	0.20
	Other viral diseases (B25-B34)	0.10
	Hypertensive diseases (I10-I16)	0.09
	Acute upper respiratory infections (J00-J06)	0.05
	Diabetes mellitus (E08-E13)	0.04
	Metabolic disorders (E70-E88)	0.03
	Injury to unspecified body region (T14)	0.03
	Other acute lower respiratory tract infections (J20-J22)	0.03
	Other soft tissue disorders (M70-M79)	0.03
	Other back disorders (M50-M54)	0.02
**Aggregation level 3**		
	Contact and exposure (suspected): communicable diseases (Z20)	0.17
	Viral infection of unspecified location (B34)	0.08
	Essential (primary) hypertension (I10)	0.07
	COVID (U07)	0.05
	Diabetes mellitus type 2 (E11)	0.03
	Injury to unspecified body region (T14)	0.03
	Disorders of lipoprotein metabolism and other hyperlipidemias (E78)	0.02
	Acute rhinopharyngitis (J00)	0.02
	Dorsalgia (M54)	0.02
	Overweight and obesity (E66)	0.02

**Figure 4:**
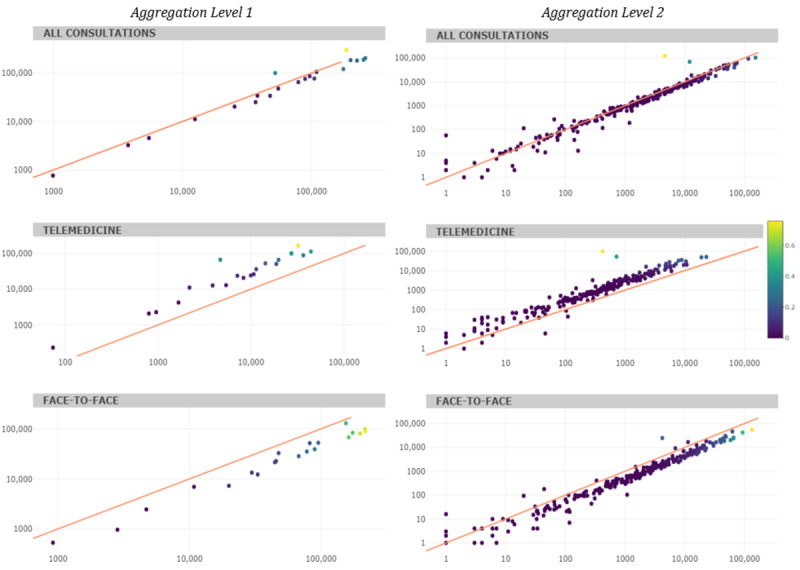
Primary care consultations before (in 2019) vs during (in 2020) COVID-19 (absolute frequency, log scale) according to *International Statistical Classification of Diseases, tenth revision*, codes. Each point corresponds to an *International Statistical Classification of Diseases, tenth revision*, diagnostic code. The 45° line indicates the identical frequency of diagnostic code use between periods; points above indicate the set of diagnostic codes that have been visited relatively more in 2020 and vice versa. The color of the dot indicates the value of the variation index (the higher, the more important the year-on-year variation).

The number of diagnostic codes decreased slightly (Figure 4), and those for telemedicine and face-to-face visits increased and decreased, respectively. Aggregation level 2 analysis shows that the diagnostic codes with the largest increase in 2020 were those related to COVID-19 (*ICD-10* codes Z20-Z29: 2.540%), along with codes related to economic and housing problems (*ICD-10* codes Z55-Z65: 44.40%). Visits with most of the other diagnostic codes decreased in 2020 relative to those in 2019. Visits for chronic pathologies such as arterial hypertension (*ICD-10* codes I10-I16: –32.73%) or diabetes ( *ICD-10* codes E08-E13: –21.13%), but also those for obesity (*ICD-10* codes E65-E68: –48.58%) and bodily injuries (T14: –33.70%) were reduced. The results show year-on-year variations (in absolute terms, an average of 12%).

The frequencies of diagnoses (listed according to the highest variation index) in face-to-face and non–face-to-face visits were systematically negative (Table 4) and positive (Table 5), respectively. It should also be noted that none of the face-to-face visit code groups were offset by those of the non–face-to-face group (ie, the largest reductions in the former were not offset by the largest increases in the latter).

**Table 4:** Top 5 in variation index of diagnostic use in face-to-face visits (aggregation level 2).

*ICD-10*^a^ diagnostic code	Total, n (%)	2019, n (%)	2020, n (%)	Interannual variation, %	Variation index
Diseases of the blood and blood-forming organs and disorders of the immune mechanism (D50-D89)	27,197 (1.03)	19,912 (1.12)	7285 (0.83)	–63.41	0.14
Mental, behavioral, and neurodevelopmental disorders (F01-F99)	128,611 (4.85)	89,415 (5.05)	39,196 (4.47)	–56.16	0.13
Diseases of the ear and mastoid process (H60-H95)	68,215 (2.57)	45,515 (2.57)	22,700 (2.59)	–50.13	0.13
Diseases of the digestive system (K00-K95)	96,028 (3.62)	67,589 (3.81)	28,439 (3.24)	–57.92	0.11
Congenital malformations, congenital deformities, and congenital chromosomal anomalies (Q00-Q99)	7107 (0.27)	4668 (0.26)	2439 (0.28)	–47.75	0.10

^a^*ICD-10*: *International Statistical Classification of Diseases, tenth revision*

**Table 5:** Top 5 in variation index of diagnostic use in non–face-to-face visits (aggregation level 1).

*ICD-10*^a^ diagnostic code	Total, n (%)	2019, n (%)	2020, n (%)	Interannual variation, %	Variation index
Factors influencing health status and contact with health services (Z00-Z99)	199,464 (15.68)	32,146 (11.20)	167,318 (16.99)	420.49	0.19
Signs, symptoms, and abnormal test results not elsewhere classified (R00-R99)	128,214 (10.08)	27,092 (9.44)	101,122 (10.27)	273.25	0.11
Diseases of the musculoskeletal system and connective tissue (M00-M99)	127,293 (10.01)	27,328 (9.52)	99,965 (10.15)	265.80	0.10
Endocrine, nutritional, and metabolic diseases (E00-E89)	157,663 (12.40)	44,155 (15.38)	113,508 (11.53)	157.07	0.10
Certain infectious and parasitic diseases (A00-B99)	71,620 (5.63)	4670 (1.63)	167,318 (16.99)	1333.62	0.09

^a^*ICD-10*: *International Statistical Classification of Diseases, tenth revision*

Finally, for children, adolescents, and adults, there were decreases in consultations for respiratory infections; ear, nose and throat pathologies; and skin problems. In adults and older adults, there were overall decreases in visits for chronic pathologies, especially those for arterial hypertension and diabetes. Decreases in visits for some pathologies were counterbalanced by an increase in telemedicine visits—among adults, for back disorders, soft tissue disorders, upper respiratory tract infections, and anxiety, and among older adults, different types of neoplasms. By sex, no differences were observed in the frequency of diagnoses (Multimedia Appendices 1-3).

## Discussion

### Principal Findings

We analyzed the differences in distinctive characteristics of care before and after the outbreak of the COVID-19 pandemic in the context of primary care in Catalonia. As expected, the increase in the frequency of visits with diagnostic codes related to *contact with or suspected exposure to SARS-CoV-2* stands out, but those related to *housing, employment and psychosocial factors* (*ICD-10* codes Z55-Z65) are also noteworthy. On the eve of a foreseeable economic crisis, this fact underlines the importance of social determinants of health and population health management when planning reconstruction policy for the period after the pandemic, as it is well known that vulnerable population groups were the worst affected by the economic crisis [19]. The change in the pattern of visits is also visible in 2 other aspects. First, the number of visits in the nursing service increased (10.54%), while those in general medicine visits decreased (–4.27%). Second, the increasing use of non–face-to-face care tools since the start of the pandemic has served a wider and more representative cross-section of the population (traditionally underrepresented groups, including low-income individuals and patients residing in the poorest areas of the country, ie, rural areas) [12]. For this reason, since the outbreak of COVID-19, and due to the role of primary care in the management of sick leave, there seems to have been a reduction in the clinical complexity of patients seen, while patients with socioeconomic problems (eg, unemployment) have increased. Visits associated with diagnostic codes for chronic pathologies (mainly endocrine and cardiovascular diseases) have been the most affected, with a decrease in frequency that has not been counterbalanced by the increase in telemedicine.

Opportunities abound for quantifying the impact of the pandemic, in addition to analyses presented herein, on service delivery and citizens’ health in order to anticipate and plan for changes in demand for health services in the coming months or years. It would be necessary to study whether, due to the lack of testing (eg, routine analysis), prescriptions performed by clinicians have been more pharmacological; which visits that were previously made in person are now conducted using telemedicine; to what extent the reduced intensity of care for chronic disease management has affected the health of citizens [6]; and whether mental health (note that the amount of this type of visit have been reduced less than the rest) will be the next pandemic [20] in order to reprioritize a care model in Catalonia which, in the opinion of the authors, was already suffering a lot of health care pressure before COVID-19.

Despite the crisis caused by the pandemic, the changes that have been made to address the crisis may improve future health systems. Big data analysis tools allow exhaustive, systematic, agile evaluation. Given the preeminence and role of primary care as the gateway to the public health system in Catalonia, the information derived from primary care information registers is very appropriate for monitoring variations in the provision of health services. This is demonstrated by the fact that the results of this study quantify reductions in the intensity of some types of visits that are consistent with our expectations. Future avenues of research are the sensitivity of information system records in predicting the severity of the situation.

### Limitations

It is worth mentioning that the 2 samples that were compared were not perfectly symmetric, insofar as the start of the COVID-19 outbreak did not coincide exactly with end and beginning of calendar years. Thus, 2020 data reflect, in part, some weeks or months of normal health care, showing some bias in favor of non–COVID-19 visits. In addition, we used *ICD-10* diagnostic codes as a proxy for the reason for visits; though it is a useful variable due to its systematic collection, it is not perfect. Moreover, while it is true that in hospitals there is a fairly exhaustive control in the coding of these variables due to the implications for billing, this is not the case in primary care, so these data may be less well coded. The circumstances caused by the pandemic may also have affected the overall quality of the data (eg, due to newly established teleworking tools among health professionals).

### Conclusions

The disruption in the primary care model in Catalonia has led to an explosive increase in the number of non–face-to-face visits. There has been a reduction in the number of visits for diagnoses related to chronic pathologies, obesity, bodily injuries, and respiratory infections, possibly as an indirect effect of COVID-19 prevention measures. Instead, diagnostic visits for socioeconomic and housing problems have increased, which emphasizes the importance of social determinants of health in a pandemic context.

Big data analysis can help to inform health planners in their decision making to focus on the least treated diseases during the pandemic. The availability of information enables comprehensive and reliable monitoring of the health system to be undertaken. In extraordinary situations, such as those created by the COVID-19 pandemic, and in view of the necessary emergency plan that will have to be carried out to compensate for the onslaught of clinical activity caused by COVID-19, big data analysis is even more relevant.

## Appendix

Multimedia Appendix 1 HTML (graphs).

Multimedia Appendix 2 EXE file with interactive plots.

Multimedia Appendix 3 Variation Index formula.

## Abbreviations

ICD-10: *International Statistical Classification of Diseases, tenth revision*
