# Supplementary figures and images for: Characterization and Identification of Variations in Types of Primary Care Visits Before and During the COVID-19 Pandemic in Catalonia: Big Data Analysis Study
Source: J Med Internet Res. 2021 Sep 14;23(9):e29622. doi: 10.2196/29622 (PMC8767991; doi:10.2196/29622)

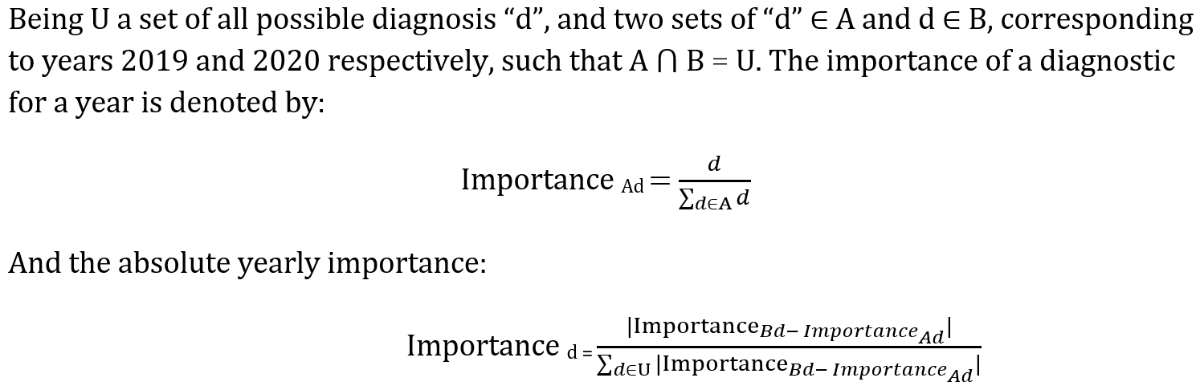

Supplement: Multimedia Appendix 3 [file jmir_v23i9e29622_app3.png]
